# An extensive sunflower dataset representation for successful identification and classification of sunflower diseases

**DOI:** 10.1016/j.dib.2022.108043

**Published:** 2022-03-13

**Authors:** Umme Sara, Aditya Rajbongshi, Rashiduzzaman Shakil, Bonna Akter, Sadia Sazzad, Mohammad Shorif Uddin

**Affiliations:** a Department of Computer Science and Engineering, National Institute of Textile Engineering and Research, Dhaka, Bangladesh; b Department of Computer Science and Engineering, Daffodil International University, Dhaka, Bangladesh; c Department of Computer Science and Engineering, Jahangirnagar University, Dhaka, Bangladesh

**Keywords:** Agriculture, Sunflower dataset, Computer vision, Deep learning

## Abstract

Sunflowers are agricultural seed crops that can be used for essential edible oils and ornamental purposes. This cash crop is primarily cultivated in North and South America. Sunflower crops are prone to various diseases, insects, and nematodes, resulting in a wide range of production losses. Digital image processing and computer vision approaches have been widely utilized to categorize and detect plant diseases including leaves, fruits, and flowers over the last few decades. Early diagnosis of infections in sunflowers helps to prevent them from spreading throughout the farm and reducing financial losses to the farmers. This article offers a resourceful dataset of sunflower leaves and flowers that will help the researchers in developing effective algorithms for the detection of diseases. The dataset contains healthy and affected sunflower leaves and flowers with downy mildew, gray mold, and leaf scars. The images were captured manually between 25^th^ to 29^th^ November 2021 from the demonstration farm of Bangladesh Agricultural Research Institute (BARI) at Gazipur in cooperation with its one domain expert when the sunflower plants were about to bloom and the maximum diseases can be found. The dataset is hosted by the Department of Computer Science and Engineering, National Institute of Textile Engineering and Research (NITER), Bangladesh and freely available at https://data.mendeley.com/datasets/b83hmrzth8/1.

**Specification Table**

**Table:** 

Subject	Computer Science
Specific subject area	Image identification, classification, image analysis and computer vision
Type of Data	Images
How the data were acquired	Sunflower cultivation sites are initially chosen by surveying areas where the land is more conducive to growing sunflowers. We decided on the demonstration farm of Bangladesh Agricultural Research Institute (BARI) at Gazipur, where a vast acre of land is being presented to grow sunflower crops. At the end of the month November (25^th^ to 29^th^), 2021, we found a large number of images of diseased sunflower leaves and flowers, as well as healthy plants. Finally, with the help of an expert, we worked on the ground to acquire raw images using a Digital Powershot Canon camera.
Data format	Raw JPG
Description of Data Collection	Previously, no such image samples had been used in any experiment. The images were acquired by us manually with the assistance of a domain expert from Bangladesh Agricultural Research Institute (BARI) at Gazipur.
Data source location	**Location:** Bangladesh Agricultural Research Institute (BARI) **Zone:** Gazipur, Dhaka **Country:** Bangladesh
Data Availability	**Repository name:** Mendeley Data **Data identification number (permanent identifier, i.e.,** **DOI number):** 10.17632/b83hmrzth8.2 **Direct link to the dataset:** https://data.mendeley.com/datasets/b83hmrzth8/1

## Value of the Data

• In a hyperspectral sequence of images, this dataset allows naked eye-tracking sunflower plant abnormalities. As a result, it enables researchers to detect the diseases in sunflower leaves and blooms early.
• Collected data can be used to develop, train, test, evaluate, and compare various machine learning and deep learning algorithms for diagnosing diseases based on visual attributes.
• The images are snapped from a larger perspective (at leaf and blossom levels). This will help the plant physiologists for further analysis to compare the disease structures of local sunflower plants to that of other variants of sunflowers found mainly in America and its environs.
• This data was collected in a natural atmosphere with inhomogeneous weather and lighting conditions. As a result, identifying abnormalities through the naked eye might be challenging for researchers.
• Early identification and diagnosis of diseases through research outcomes might encourage farmers to make massive sunflower production that can add value to the national economy.

## 1 Data Description

A sunflower plant, commonly known as a seed of productive oil crop. The disease of the sunflower plants is a crucial factor in lowering flower and fruit (seed) yields and their application in a variety of businesses. In these circumstances, this dataset can be a state-of-art mentor in developing algorithms for early identification and diagnosis of sunflower diseases in the agricultural domain.

Sunflowers are affected at the flower, leaves, stems, and roots by almost three dozen diseases caused by bacteria, fungus, nematodes, parasites, viruses, etc. [1]. Some of the bacterial diseases are apical chlorosis, bacterial leaf spot, bacterial wilt, head rot, etc. In contrast, the fungal diseases are botrytis head rot, known as gray mold, downy mildew, charcoal rot, stem spot, rust, white rust, yellow rust, powdery mildew, phoma blight, septoria, leaf scar, etc. However, in our region three fungal diseases gray mold, downy mildew, and leaf scars are very frequent. With this view, the current dataset is concentrated on these three fungal diseases. This article includes a data set containing a total of four hundred and sixty-seven (467) original images and sixteen hundred and sixty-eight (1668) augmented images prepared from the original images of healthy and disease-affected sunflower leaves and blooms. The distribution of this dataset is depicted in Table 1. All the images are captured manually by a digital camera with the assistance of a domain expert from Bangladesh Agricultural Research Institute (BARI) at Gazipur. All the images hold a constant width and height of 512×512 pixels sequentially. Table 2 contains the details of the dataset for each individual class of diseases.

**Table 1:** Class wise dataset distribution.

Class Name	Number of Original Images	Number of Augmented Images
Gray Mold	72	398
Downy Mildew	120	470
Leaf Scars	141	509
Fresh Leaves (Disease-free)	134	491

**Total**	**467**	**1668**

**Table 2:** Brief description of the dataset.

Disease Name	Description	Visualization
		
Gray Mold	Gray mold is a fungus that attacks the flowers, leaves, and stems of a wide range of flowering plants. Sunflower leaf and fruit indicators are small, bent, and under dangerous places with dark squares. Gray mold, also known as botrytis blight, rots or hinders the opening of sunflower buds. It also causes the mature flowers to discolor. The silvery-gray spore masses of the mold are a telling sign. Yellow patches emerge on the backs of the baskets as a symptom of the disease, and the tissue is eventually covered in a reddish-gray mold. The mold is then applied to the entire basket. The basket rots after 7 to 10 days. The seed coat becomes permeable and mottled as the disease progresses. Sclerocytes grow on the seeds' surface as well as inside them [2].	
		
Leaf Scars	Sunflower leaf scars caused by Septoria helianthi fungus are widespread and don't pose a substantial threat. As a result, when combined with other diseases, it can seriously harm the plant's growth. On the leaves, unevenly shaped green to yellow dots develops. Inside the spots, black dots appear, which signify the fungus' fruition. The tissues that are damaged perish, and the leaves wilt.	
		
Downy Mildew	Downy mildew caused by Plasmoparahalstedii is found in nearly every country where sunflowers are planted. In the presence of existing control procedures, the global impact on yield has been estimated to be 3.5 percent of commercial seed output. In comparison, yield loss in contaminated fields can be as high as 100 percent [3].	
		
Disease-free	Sunflower leaves are triangular to heart-shaped in form and have serrated borders. Hairs cover both surfaces of the leaves, which are 4 to 12 inches long. Stems are strong and upright, ranging in height from 3 to 12 meters, and are generally purple with green dots, densely hairy, and branch towards the plant's top half.	

## 2 Experimental Design, Materials and Methods

### 2.1 Camera specification

The data was collected using a Canon Powershot Digital Camera (SX540) having 20.3 MP with a CMOS sensor and 20.3 MP of effective pixels. The sensor is 1/2.3 inch with an ISO rating of 80-1600. Each capture (in the JPG format) is assigned as an RGB color spectrum, with 256 shades for every RGB layer and 8 pixels for every shading layer. The measuring lens covers 50×50×50 along with a focal length ranging from 0 cm to infinity (W).

### 2.2 Preprocessing

**Fig. 1:**
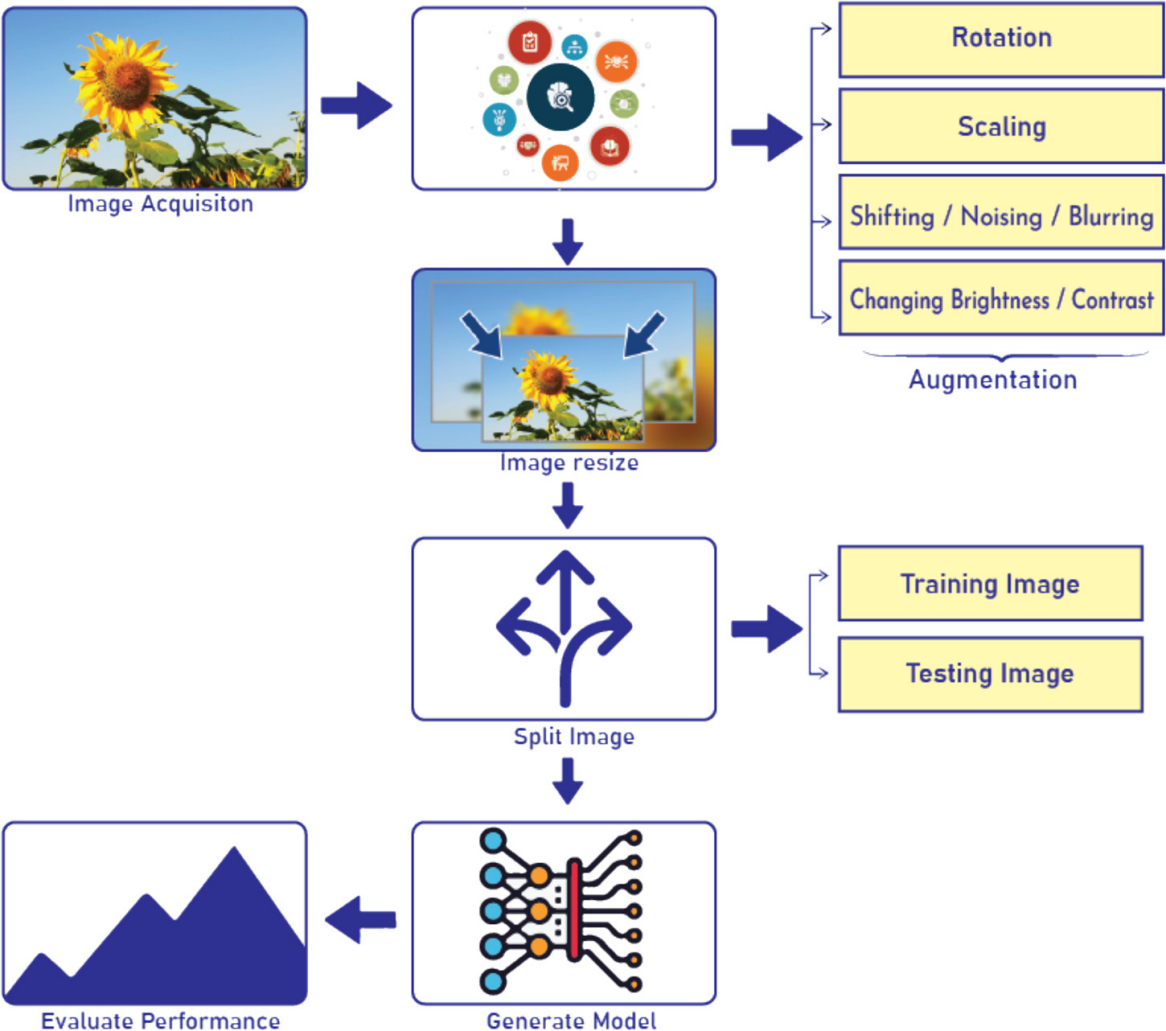
Generic processing steps of sunflower disease classification.

**Fig. 2:**
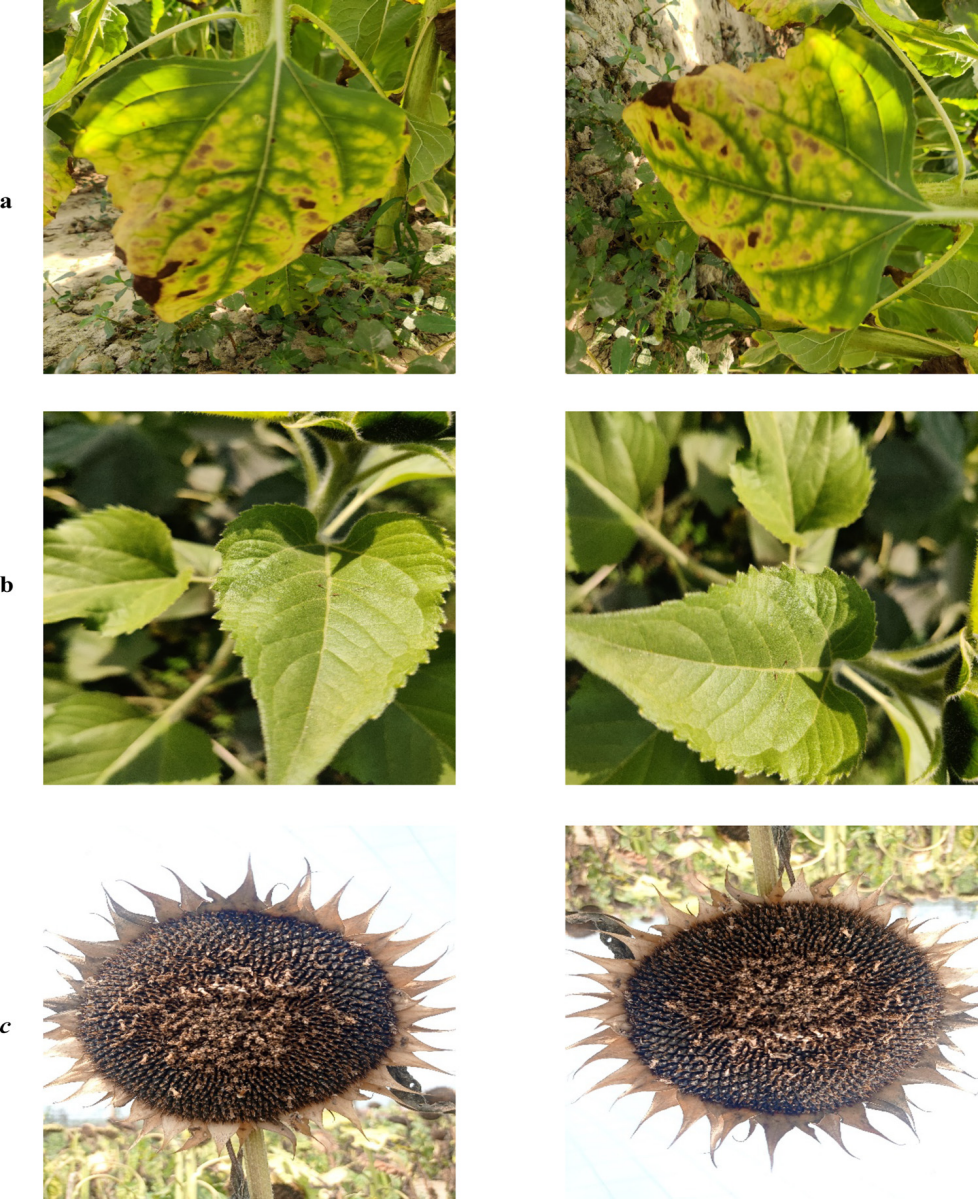

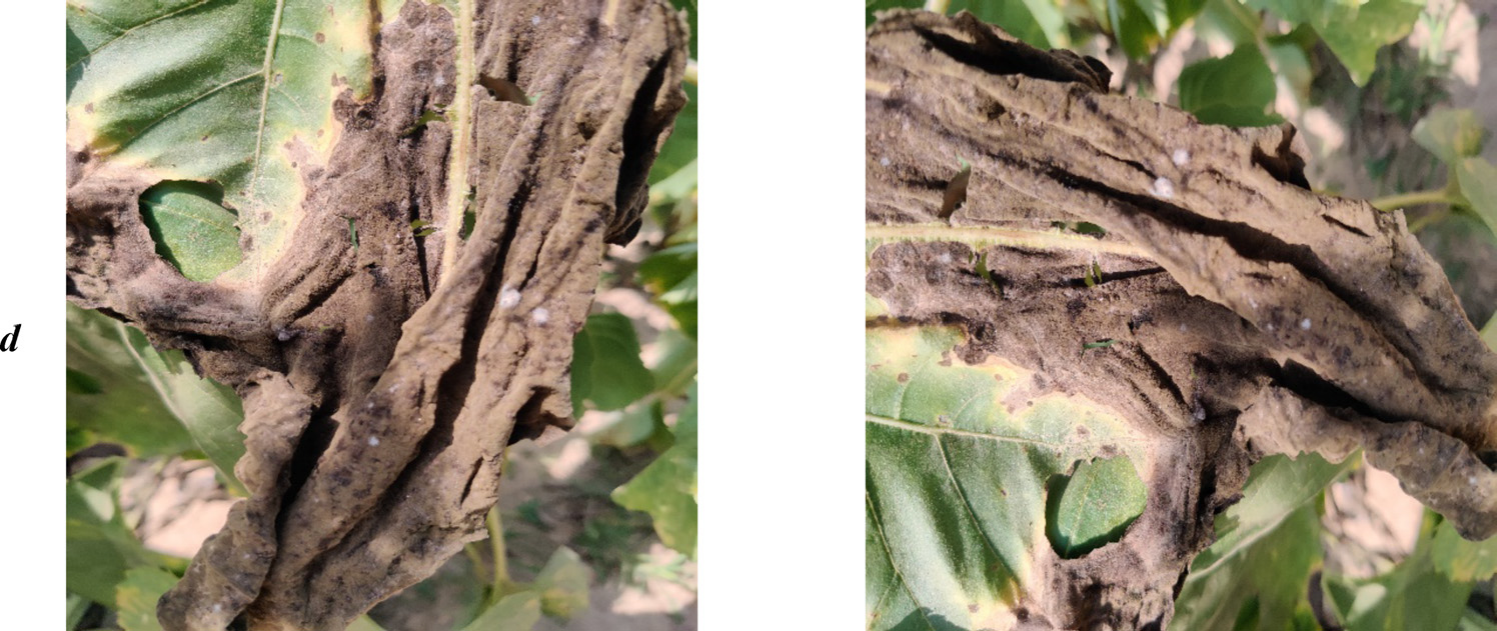
Sample of augmented images (right) from the original image (left) with a) Downy Mildew b) Disease-free c) Gray Mold and d) Leaf Scars.

To successfully train the dataset, we propose a traditional deep learning model with a view to get a state-of-the-art outcome. Now, this worthy process crosses over five major steps such as (a) Image Augmentation, (b) Resizing, (c) Splitting Images, (d) Model Generation, (e) Performance Evaluation and is visualized in Fig. 1. As the acquired images are random clicks, we need to organize these images into a uniform size and shape. In the image augmentation process, images are augmented on a large scale as we know a huge amount of data is required to train a deep learning model. So, model performance is also improved according to the increased amount of data. Here a general augmentation process is done through two ways, such as position and color alterations. Position augmentations are done through random rotation, scaling, shear, exposure, cropping, shifting, noising, and blurring, etc. On the otherhand, the color augmentation are done through changing brightness, contrast, saturation, and hue, etc. In this dataset, rotation, scaling, and shearing are performed to do the augmentation. Samples of augmentation for this sunflower dataset are presented in Fig. 2. The parameters have been used in this process are: rotation with 90°, 60° and 45°, width_shift_range is 0.1 and height_shift_range is 0.1 and shear_range is 0.1. A function named fill_mode() in the Keras library in Python has been used to execute these parameters for augmentation. Nearest-neighbor interpolation, Bilinear interpolation, and Bicubic interpolation are some standard rescaling techniques implemented successfully. Histogram equalization is another technique that has been implemented to enhance the contrast of an image. All images must be scaled to constant size and pixels before being input to the neural network model. Hence we resize the augmented images into the dimension of 512×512 pixels. Then the resized images are split into training and testing images. The sunflower dataset is provided here to perform categorical classifications for the early diagnosis of three most frequent fungal diseases that will find wide real-life applications. Besides a generic flow process is presented in Fig. 1 to recognize the diseases. In the next step, the expected model will be generated and the performance of the model will be estimated.

## Ethical Approval (involvement of animals)

This article does not contain any studies with animals performed by any of the authors.

## Ethical Approval (involvement of human subjects)

There are no studies involving human participants done by any of the authors in this article. The datasets used in the article are open to the public. For the usage of these datasets, proper citation rules should be maintained.

## CRediT Author Statement

**Umme Sara:** Conceptualization – original draft preparation; **Aditya Rajbongshi:** Methodology, Software; **Sadia Sazzad:** Data curation and Visualization; **Mohammad Shorif Uddin:** Supervision, Reviewing, and Editing; **Rashiduzzaman Shakil:** Validation; **Bonna Akter:** Writing and Investigation.

## Declaration of Competing Interest

The authors declare that they have no conflict of interests from any competing financial interests or personal relationships that could have appeared to influence the work reported in this paper.

## Data Availability

Sun Flower Fruits and Leaves dataset for Sunflower Disease Classification through Machine Learning and Deep Learning (Original data) (Mendeley Data).
